# Inaugural Address, Delivered at a Meeting of the Medico-Botanical Society of London, Held February 16th, 1831; Philip Henry Earl Stanhope, President, in the Chair

**Published:** 1833-04

**Authors:** Gilbert T. Burnett

**Affiliations:** Professor of Botany to the Society, and Lecturer in the School of Physic, St. George's Hospital, &c. &c. &c.


### INAUGURAL ADDRESS.

*Inaugural Address, delivered at a Meeting of the Medico-Botanical Society of London, held February* 16*th*, 1831; Philip Henry Earl Stanhope, *President, in the chair.* By Gilbert T. Burnett, Professor of Botany to the Society, and Lecturer in the School of Physic, St. George's Hospital, &c. &c. &c.

(Continued from page 100.)

Of the energies of our native plants the herbalists of a former age were doubtless too easily persuaded; their experience could have scarcely justified the encomiums they lavished upon many; at least, our observations will not permit us to corroborate their praises: but, however ready we may be to allow (and no one is more ready than myself,) that the curative powers of many plants have been greatly exaggerated, and that ignorance and superstition have attributed effects to other things than causes, and have often thus ascribed to various vegetables, virtues which they have not, and never had; still I can hardly think that all their asseverations could be based in error; even the greatest lies are generally founded on some little truth; indeed, without it, they scarcely could be framed. And when we consider how truly significant are the common names of plants, such as woodbine, cleavers, sea-thongs, catch-fly, rest-harrow, choke-pear, and many others; when we reflect how justly descriptive is our native nomenclature of many of the properties of the plants to which the rustic names belong, such as bitter-sweet, hedge-mustard, water-pepper, fool's-parsley, hen-bane, dog-bane, flea-bane, cow-bane, bug-bane, fish-bane, bane-berries, bane-wort, deadly nightshade, and so on, shall we precipitately conclude that the malevolent terms alone are akin to truth, and that balm, and eye-bright, thyme or strength, worm-wood, tetter-wort and quinsy-wort, scurvy-grass, fever-fuge, and a multitude of others, are all unmeaning appellations, because forsooth the ancient cure-all, all-heal, all-good, long-life, and live-for-ever, seem, when misunderstood, somewhat hyperbolical expressions.

"Sell your coat," says the Italian proverb, "and buy betony:" wherefore should this plant have been formerly so much esteemed? So little is it now regarded, that we might almost say it is despised. This question, however, will not come alone: for the same may be pertinently asked of our dysentery, uvula, and tooth-worts, of our mad-worts, liver-worts, and gout-worts. But, even granting that some, or many, or even all, our health-promising plants, have no right to their appellations, i. e. exhibit no sensible properties when taken, (which, however, I am far from presupposing,) will it be no advance in knowledge to ascertain the fact of their inefficiency? Will it not avail us much to know that such and such plants do *not* possess either medicinal or poisonous powers, instead of continuing in unenviable doubt? for such as are unfit for physic will afford harmless, and often most nutritious food. Hence I am far, very far, from underrating such negative knowledge; and I am sure I am as far from overvaluing its importance, when I state that, in the treasury of science, it becomes positive information: just as in that language which lives alone to learning and seems to science sacred, two negatives have an affirmative, or, according to their relative situation, more than an affirmative meaning. For example, *non-nihil* and *non-nunquam* simply reverse their separate import, and may be translated *somewhat* and *sometimes*, while *nihil-non* means *every thing*, and *nunquam non* means *always*. So, as is symbolized in this apposite illustration, negations in experimental researches, when duly appreciated and properly employed, become most important positive affirmations.

But to return to the point whence this episode sprang, and to the path whence a favorite topic has so long seduced us. There are several circumstances connected with the history of quinia and cinchonia, the proximate principles of the Peruvian barks, to which, might I so far presume, I could much wish to invite the attention of my colleagues, the learned professors of Materia Medica and Chemistry; for to my mind they require much further elucidation, and many experiments seem wanting to ascertain correctly the properties of these barks, and the principles they respectively afford. The quilled or pale-brown bark was a few years since so much more highly prized than the yellow, that it was sold for sixteen shillings a pound, whilst the other would scarcely fetch a crown; and yet quinia, now so much esteemed, and believed to be the potent principle of the Peruvian barks, is abundant in the yellow, and all but absent from the quilled. Indeed, I am informed by my friend, Mr. HENNELL, of Apothecaries' Hall, one of the most indefatigable and intelligent pharmaceutic chemists of the day, that if the specimens submitted to analysis be correctly sorted, (for, in commerce, they are generally met with mixed,) no quinia at all will be found in the quilled bark, but abundance of cinchonia; while, in the yellow bark, the quinia is abundant, and the cinchonia wanting.

Now this is a result which could not, *a priori*, have been conceived: for it is the *quinia* which is now extolled for its extraordinary curative effects; which principle, be it noted, is absent, or all but absent, from the quilled bark, so lately prized for similar powers; while the *cinchonia*, which abounds therein, is disesteemed: and, on the other hand, as a counterpart to this paradox, *the yellow* bark, so long neglected, yields abundantly, almost exclusively, *quinia*, the present fashionable tonic, whilst it contains none or very little of that other principle *cinchonia*, to which all the former cures of its compeer, the lance-leaved bark, must be attributed ; it being, as already stated, in an equal degree devoid of quinia.

In the *red* bark both *cinchonia* and *quinia* are found, but in much smaller relative proportions. It is likewise a circumstance worthy notice, that some of the other species of cinchona, specimens of the bark of which occasionally reach us, are said to afford these principles more abundantly than any of the officinal ones; and it is also probable, as shewn by Dr. HANCOCK, that other genera may be more advantageously used as febrifuges than cinchona.

Now, from these premises, we cannot avoid the conclusion that further experiments and investigations, both botanical, chemical, and medical, are much required: for if the quilled bark really did cure ague, and of this there can be no doubt, and if in it there is little or no *quinia*, but *cinchonia* is present in abundance, either its native quinia must be converted into cinchonia, or the neglect with which cinchonia is treated can scarcely be its due. Again, the merits of quinia may perhaps have been too much extolled; or it is not impossible that the red bark, which contains the two principles in question, or a preparation or formula comprising both quinia and cinchonia, whencesoever extracted, may be found the most efficient mode of administering relief in ague.

But these are problems requiring much attentive consideration; and experience must prove whether the former confidence in quilled bark, or the present trust in quinia, be most correct. Probably cinchonia is esteemed too lightly; it demands a series of experiments to ascertain its medicinal effects. This is, however, but one of many points equally requiring investigation, and which must be more fully entered on hereafter; and it is only at this present time glanced at, to show how much and in how many ways the exertions of our Society are wanted; how much and in how many ways its members can benefit the world at large. For it is not improbable that many of these proximate principles, now considered as different and distinct, may be hereafter found to be similar in effects, if not absolutely the same in constitution, just as all the old salts of plants, viz. of wormwood, centuary, broom, and others, have been proved to be one and the same, viz. subcarbonate of potash.

The extraction of the proximate principles of plants, of those especially on which their peculiar properties depend, is doubtless a triumphant step in Phytochymics, or Vegetable Chemistry, and is another point to which I must briefly draw your attention; as it is a discovery which promises to open a new era in science, and to work a complete revolution in the armoury of physic. To the superficial observer it may seem that these discoveries, though undoubtedly of very modern date, had been long obscurely adumbrated, as it had for years been known that the stimulating powers of many plants, as of the Cruciferæ, for example, were destroyed by simple exsiccation; that the poisonous principles of Mercurialis perennis might be extracted by boiling in water, when it becomes a palatable and harmless esculent; that the virulent qualities of the Cassava are separated by heat, and that, after roasting, it is esteemed an admirable and nutritious food. This knowledge, however, differed widely from that which has enabled us to reduce and exhibit, in a constant and determined form, quinia and cinchonia as the active and proximate principles of bark; salicine from willows; veratria from colchicum and white hellebore; morphia, narcotine, and meconic acid, from opium; and so forth. Great caution, almost a jealous caution, is, nevertheless, required in watching both the extraction of these principles and the records of their effects: for it is not impossible, nay, it is far from being improbable, that some of these substances are products rather than educts of the chemist's operations; just as sugar may be produced from rags, tan from saw-dust, vinegar from wood, oxalic acid from offal, and so on. Not that the mere fact of such transmutations would render the substances produced a whit less valuable than if they were educed; their value must depend upon their intrinsic worth; and it may be far more advantageous, under certain circumstances, to resort to the crucible of the chemist, than to the laboratory of nature, for many of them. We know that wood consists elementarily of charcoal and water; that starch, and gum, and sugar, are similar in their elements likewise: and that they only differ from each other in their relative definite proportions: we know that nature, by varying the atomic ratios of these few simple elements, changes, as in the ripening of fruit, tasteless lignin into powerful acids, which in their turn become converted into sugar, often impregnated with peculiar essential aromatic oils; and thus, in plants, her living retorts, Nature, from watery sap and atmospheric air, so modifies the union of three simple bodies as to form gum, sugar, starch, lignin, resins, oils, acids, alkalies, and alkaloids, with numerous other proximate principles in abundance, of every kind and in every variety; and this by merely varying the relative properties of the same simple constituents, oxygen, hydrogen, and carbon,—these three, for others are seldom and sparingly employed. We also know that in some instances our laboratories can imitate the vegetable alembics, or render their processes subservient to our designs. Thus, in malting barley, germination is suddenly checked when the starch of the grain has become converted into sugar; and by heat the same starch can be transmuted into gum. It is, therefore, not impossible that many of the more active vegetable principles may be in like manner formed, the more scarce from the more abundant. That some of the newly-discovered bodies, at present, for convenience, called proximate vegetable principles, such as caffein, asparagine, piperine, and so on, are not *educts*, but *products*, is more than probable, when we find that they do not exhibit the specific effects peculiar to the substances from which they are obtained. Thus we learn from Mr. Brande, that coffee is narcotic, but caffein, as that celebrated chemist proved by experiment on his own person, has (at least in scruple doses,) no sensible narcotic powers; and the effects of morphia bear no proportion to the effects producible by the quantity of opium consumed in its formation. Again, asparagine does not communicate to urine the peculiar odour which asparagus is invariably found to do; and, furthermore, piperine, the so-called proximate principle of pepper, is entirely destitute of pungency and heat. These, then, and many others, will probably be hereafter shown to be products of the chemist's operations; just as beer and wine are not, as they are often called, the juice of barley and the grape, but are produced, not educed, therefrom, by fermentation; and alcohol, including brandy and spirit of every kind, is not educed, but produced, from wine and sweetwort by distillation, or formed by condensing the vapours which rise from bread while baking.

It may be, then, (and I repeat it for the sake of the impression which a repeated proposition is designed to make,) it may be found hereafter more profitable to form many of these substances now known as the proximate principles of vegetables, directly from their elements, or indirectly from the mutation of common and abundant matters in the laboratory of the chemist, than to procure them intermediately from plants, the living elaboratories of nature; just as oxalic acid can be made from hair, gristle, and such like refuse matters; or produced by the distillation of sugar with nitric acid, instead of being educed from the Oxalis acetosella, corniculata, &c., where it naturally exists; or as prussic acid is now obtained from prussian blue, which can be made from almost any offal, at an amazing diminution of expense to what it could be educed from bitter almonds, cherry-laurel leaves, plum and peach kernels, &c. where the acid naturally exists, and which were once esteemed its only sources. Indeed, one great object of the vegetable world would seem to be, to anticipate the arts, and to provide numerous comforts and conveniences, such as clothes and shelter, nutritious food, and efficient medicines, for man, long before he was able to provide any for himself; and not for man only, but for the whole animal creation. Of this vegetable physiology affords numerous interesting illustrations: and although at present we contemplate the processes of nature but as through a glass, darkly; although we see but in part, and know but in part; still thus far we can perceive, that, whether we sleep or whether we wake, whether we rest, or whether we toil, these indefatigable servants are labouring for our advantage; that they are ever actively preparing and purveying sugar, starch, gluten, gum, cotton, wood, flax, hemp, and many other substances, with an alacrity, a perseverance, and a certainty, which would be truly astonishing, were we not surrounded by such natural miracles, and did we not live in a world of wonders. Many of these works will doubtless ever remain inimitable by human art; to bounteous nature we must ever be indebted for her most precious gifts. We never can hope to penetrate the arcana of all her mysteries, although in some few particulars we can trace her course, and imitate her productions. Still, as we have already much lightened human labour by the substitution of brute strength, and superseded the use of many brutes by the adaptation of machinery, thus reclaiming much soil from the pasturage of cattle for the growth of food for man, and, by the introduction of steam and rail-roads, we shall reclaim much more; so it is not improbable that much of the labour of tilling the ground for the growth of human food may hereafter be avoided, by the immediate production of many substances from their elements, instead of deriving them intermediately through the culture of plants; or, at any rate, it is not improbable that we may so far advance in science and in art as to be enabled to convert the less into the more useful; the more into the less abundant: as wood into starch, worn-out flax, i. e. old linen, into sugar, and so on; and thus, if it were not for placing an important subject in rather a ludicrous point of view, we (in like manner as spiders and some other insects are said to devour their exuvial skins,) might hope to be enabled, after having worn out our clothes, to feed upon them, and to dine off as well as on our tables.

There is but one topic more with which at present I will intrude on your attention; but it is one which, as it most especially relates to the duties of the professor of botany, I should be inexcusable to let pass unnoticed.

Much of the obscurity attendant on the history of the effects of drugs is doubtless attributable to the careless manner in which plants have too commonly been collected and preserved, but often, still more to the errors which the ignorant, and the frauds which the unprincipled, commit. Hence, it is necessary that a continual check should be kept by the eye of science over the gatherers and venders of medicinal herbs. How often do we find briony-root substituted for calomba, mullein-leaves for foxglove, horn-beam for hops, marigold-petals for saffron, cow-parsley for hemlock, &c. Into one of our largest drug-factories there once was brought a large quantity of Œnanthe crocata, which, had not a botanic eye been casually cast upon the heap, would have been both sold and bought for conium; and had an extract, or tincture, or powder of its leaves been used, how disastrous might have been the result. This is not a solitary instance; such mistakes are unfortunately still too common: but henceforth they can less easily escape detection, since a knowledge of botany has been made a necessarily integral part of every medical education. Indeed, much as we may value the labours and researches of the physician and the chemist who investigate the medicinal properties and constituent principles of plants, the labours of both would be often of no avail, did not the botanist aid them in determining, by constant and decided characters, the very plants in which such principles are found, and to which such properties belong; as well as in what situation and under what circumstances they should grow, at what seasons and in what states they should be collected; for all these will often much affect their potency and value: e. g. how different is the Apium graveolens growing in the light to the same plant when excluded from its access? how importantly does a wet or a dry situation affect the character of many umbelliferous plants; for the dry soil which increases the aromatic properties of some, diminishes the essential powers of others, while the wet situations which impair the aroma of these, enable those to eliminate to the full their active poisons. Again: many plants similar or nearly so, and which are frequently undistinguishable to a non-botanic eye, often possess very different properties: thus, to take only the most common and familiar cases, how important it is to distinguish Æthusa Cynapium from the true culinary parsley, the Lolium temulentum from other grasses, and so forth. Thus, how vainly should we seek in Cichorium Intybus for the hepatic influence of Leontodon Taraxacum; and yet, to superficial observers, so similar in their early states are these two very distinct plants, that they are being continually and constantly mistaken for each other; in fact, they form the pons asinorum of all inceptor candidates for botanic fame.

But I feel that it would be here a work of supererogation to illustrate more fully the utter practical inutility of many of the researches of the physician and the chemist, without the botanist would labour with them. What avails it that the physician knows that opium brings sleep, lulls pain, and stays the approach of many ills; that Peruvian bark cures ague; that jalap and scammony are drastic, and rhubarb a tonic astringent purge? What avails it that the chemist by analysis can prove that the sedative principle of opium is morphia, the tonic of cinchona, quinine; or what avails it that he proclaims the equally-important facts, that certain specimens of bark submitted to his examination contain a large, and others a small, portion of one or other of the active or inert principles, without the botanist could indicate the plants which yield them, and teach to distinguish those in which the active agents the most abound, and which consequently may be the most profitably employed? Say not that the merchant or the druggist will import and prepare them to his hand: this would indeed be trusting to a rotten staff, without they were themselves instructed in that science which alone can teach to distinguish those which are found less from those which are found more efficient in their use and productive of the active agents sought. Are crops to be cultivated and cargoes to be imported on chance or bare empirical presumption, and the first notice of error be in the disappointment of the physician at the non-efficacy of his prescription, or in the vain labour of the chemist to extract the essential parts?

Besides the certain diagnoses it affords, there is another advantage of botanical system, especially of the natural system, which, although it is very much perverted and abused, holds, in my mind, a rank of no mean importance: viz. the general rule, sanctioned by experience, that plants possessing the same botanical characters often contain the same chemical principles, and produce the same medicinal effects; not always, indeed seldom, in a similar degree, but most frequently in a graduated scale. The potency being traceable in the various instances from its least to its greatest accumulation, from its lowest to its highest stages of activity. Thus, the Lactuca virosa is highly acrid, while the Lactuca sativa is bland and esculent; the Cucumis sativus an agreeable food, while the Cucumis colocynthis is a powerful medicine; the Vegetable Marrow, Gourds, and Melons, mild and innoxious, while the Momordica Elaterium is so violent in its effects as almost to be esteemed a poison. Again, the Croton Cascarilla is a grateful stomachic, while the Croton Tiglium is a most violent cathartic; the Leopard's bane may be contrasted with the artichoke, and parsley with hemlock; the Solanum dulcamara, nigrum, and tuberosum, bear hurtful berries; while those of the egg-plant and the tomato, when cooked, form a wholesome food; the pea and bean are only flatulent, while the laburnum is a poison. And furthermore, so completely are the latent properties of some plants developed in other individuals of the same species, genera, and orders, while the notorious principles of those are overshadowed or become occult in these, that, did not instances occur in different varieties of the same species, and different species of the same genera, they would seem to form more serious objections to the present distribution of the larger groups than they actually do. Thus, in one group, the Umbelliferæ, are found the venomous Conium, Œnanthe, and Cicuta, the harmless and nutritious Carrot, Parsnep, and Earth-nut, the stinking Galbanum, Opoponax, and Assafœtida, and the grateful aromatics, Cummin, Coriander, Carraway, and Dill. But, then, it should be remembered that, in the same genus, *Œnanthe*, while the roots of the species *Crocata* afford a deadly poison, those of the species *Pimpinelloides* form a wholesome food; and even in the same species, *Apium* graveolens, the cultivated Celery, is a harmless, while, in an uncultivated state, it is a deleterious plant. Once more, the milk-tree and the bread-fruit are associated with the Upas of Java; but the connexion is established between them by the figs; for, although most are harmless, one species of the genus *Ficus*, the Toxicaria, is, as its name imports, an acknowledged poison. Of these illustrations there would be no end, did time allow of the pursuit: on another occasion I may, probably, should it be sanctioned by the Council, bring the matter more at length before the Society, as it is one that requires much serious deliberation.

Vegetable physiognomy (if I may so express myself) is a study well deserving cultivation; for it is most important to be able, on looking at a plant, to affirm, on principle, this may be eaten; that is a poisonous plant; or, again, those are suspicious vegetables: such knowledge, moreover, is not only desirable to have, but easy to be attained; and its practical utility has already been, in several instances, shown under circumstances of urgent need. It is, however, a skill in vegetable physiology, a knowledge of the laws of vegetable metamorphoses, which can alone enable us to decide such points with safety; and it is one of the most delightful of all botanical praxes to work out the relation of organs apparently, but not really, different, and thus to establish the *hetero-* and *iso-morphous* characters of plants, often improperly associated or severed; and to the rudimentary state of this branch of botany may be attributed those anomalies, those apparent paradoxes, which at present form exceptions to our natural schemes. But even this power of diagnosis may be, nay, it already has been many times perverted; and men, presuming on their wisdom, when they ought rather to avow their ignorance, have supposed their knowledge of these general laws so perfect and complete, that some have not scrupled to declare it *impossible* for nature to swerve from those they have defined, and refused to believe in the possible occurrence of exceptions. Exceptions, and numerous exceptions, do, nevertheless, undoubtedly exist: these should, however, rather stimulate us to review our natural classifications, than to discard them altogether; should lead us rather to distrust our own performance, than to accuse nature for a moment of egregious error.

The true purposes and value of the natural and artificial systems, the aid they reciprocally afford each other, and the mode in which they may be most advantageously employed in the advancement of medico-botanical science, will form another very important topic, which, with the permission of the Council, I purpose submitting to the consideration of the Society. It is one that I consider of paramount importance, and the correlative utility of these two systems is a point for which I have long most ardently contended, and one which I have not neglected to inculcate, both in my lectures at the Royal Institution, and in the medical schools of Great Windmill-street and St. George's Hospital: for I am sure that either, when alone, would be a most imperfect guide; while from them in conjunction we derive a clue which will be often found to lead to discoveries of the greatest brilliancy and value. Analogy will lead to experiment, and experiment, guided by analogy, will lead us to truth.

But, to conclude. In the botanical researches of this Society, it is evident that much discretion must be exercised, so as to render complete the medicinal history of the subjects we propose to illustrate, without including in our transactions topics foreign to medico-botanical science. Hence, although your professor of botany is sensible, no one more so, of the interest and importance of anatomical and physiological, as well as systematic and economic botany; and although he would consider himself wanting in his duty to this Society, were he to neglect any of these departments, all which bear, either directly or indirectly, on each other; still he would regard himself as perverting the objects of the Society, and wandering from the designs of its institution, were he to occupy the time of the meetings, or the pages of the Transactions, by any disquisitions on mere anatomical, physiological, or systematic themes. Many of them must, of course, engage his attention, as promising to throw light on medical botany; but whenever individual research has shown them to be impertinent thereto, they cease to be objects fit for presentation here. Their value to science, however, is not the less; at other times, and in other places, if presented, according to their worth so will they be esteemed: and this seems the more necessary to be stated, lest any should precipitately conclude that general botany and medical botany are two different sciences; whereas, the one is but a part of the other, just as the fruit is but a part and production of the tree on which it grows. Hence, it must be evident that there are no means practicable of learning just so much, and so much only, of the philosophy of the vegetable world, as may be immediately applicable to the purposes of medicine, without at the same time studying the science as a whole; and this could alone efficiently be done by making it an independent subject for instruction, and enacting that an attendance on botanical lectures should henceforth become an essential part of every student's duty; for, without such knowledge, no medical education can be truly esteemed complete. The junction of botany to materia medica is absurd, as one must, of necessity, be neglected; for if, in the time allotted to the course, equal attention be paid to both, both will be imperfectly and unsatisfactorily taught. The courses must be kept essentially distinct, even when delivered by the same professor. If, in the infancy of physic, their union was tolerated, and botany studied merely because some plants afforded medicines, such an occultation no longer can be borne. Both sciences, by such a scheme, are injured, and keep each other, as it were, in thraldom. They are both sufficiently extensive and important to form separate studies.

The advantages resulting from the division of labour are too well known, and have been too long established, to need illustration here; and our Society, although akin to others, of which it would almost seem an emanation, has objects as important and distinct as those have from their antitypes. Thus, the Royal Society, which at first, and in our infant days of science, included in its fostering arms every department of philosophy, soon gave rise to other associations for the especial advancement of particular sciences: such as the Antiquarian Society, for archæological researches; the Linnæan Society, for the encouragement of natural history, whence the Horticultural, the Agricultural, the Medico-Botanical, and Zoological Societies have sprung. Thus, as science advances, each separate branch, arriving at maturity, quits the parent institution which nurtured it in youth; and hence there has arisen that brilliant galaxy of British associations, of which our country justly boasts, as the Royal Society, the Royal Society of Literature, the Royal Institution, the Medical and Medico-Chirurgical Societies, the Geological, Geographical, Astronomical, and numerous other learned associations, all striving in honourable warfare against indolence and error, all engaged in a glorious crusade to recover the holy land of science from the infidel dominion of ignorance and sloth.

Gentlemen. In attempting to develop what he considers to be the legitimate objects of your Society; in attempting to trace, as it were in outline, what he considers to be the duties of his office; your professor of botany is aware that he has very inefficiently foreshadowed the object he had in view; and yet, insufficient as the sketch avowedly is, he is sensible that, in following it practically out, he shall often have occasion, as at the present moment, to claim your indulgence for much that has been done imperfectly, your pardon for much which has been wholly left undone: and yet he doubts not that, when zeal in your service is apparent, you will kindly, from your own resources, supply that which in him seems wanting.

Your professor of botany is likewise sensible how much he must depend, how much he must be indebted to the cooperation and separate exertions of his colleagues; yet he doubts not that, from their well-known ardour in the pursuit of science, they will always befriend him in completing those accounts of medicinal plants, from time to time to be presented to the Society, and for which duties none can be more competent than themselves: for thus conjointly we may hope to be enabled to collect such ample details, and such certain records, drawn from actual observations and experiments of the principles, properties, and powers of the vegetable materia medica, as may go far towards perfecting the practice of medicine, and rendering, as HOMER says,

> "A wise physician, skilled our wounds to heal,

> More worth than armies to the public weal."

